# Supplementary material for: Killing from the inside: Intracellular role of T3SS in the fate of Pseudomonas aeruginosa within macrophages revealed by mgtC and oprF mutants
Source: PLoS Pathog. 2019 Jun 20;15(6):e1007812. doi: 10.1371/journal.ppat.1007812 (PMC6586356; doi:10.1371/journal.ppat.1007812)
Supplement: S1 Fig — J774 macrophages were infected with PAO1 wild-type strain expressing GFP. Time lapse imaging was started at 1.5 hrs post-phagocytosis. Cells were maintained in DMEM supplemented with gentamicin, at 37°C and 5% CO2 throughout imaging. White arrows point at infected cells that undergo lysis, whereas black arrows indicate uninfected cells that do not lyse. Images were taken between 1.5 hrs and 3 hrs post-phagocytosis as shown on the panels. Scale bar is equivalent to 10 μm. (PDF) [file ppat.1007812.s001.pdf]

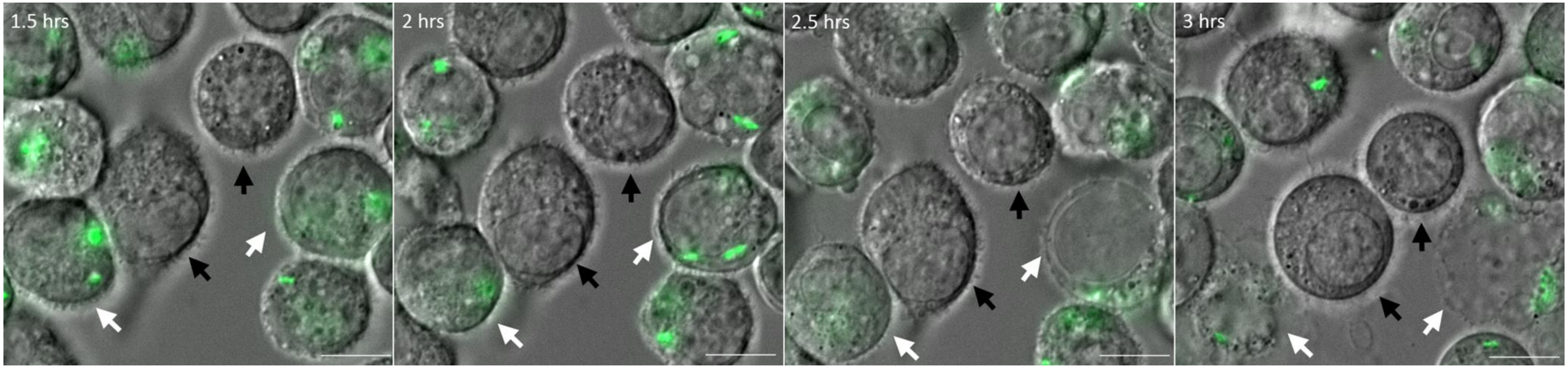

S1 Fig. Live imaging of macrophages infected with *P. aeruginosa*. J774 macrophages were infected with PAO1 wild-type strain expressing GFP. Time lapse imaging was started at 1.5 hrs post-phagocytosis. Cells were maintained in DMEM supplemented with gentamicin, at 37 °C and 5% CO_2 throughout imaging. White arrows point at infected cells that undergo lysis, whereas black arrows indicate uninfected cells that do not lyse. Images were taken between 1.5 hrs and 3 hrs post-phagocytosis as shown on the panels. Scale bar is equivalent to 10 µm.
