# Supplementary material for: Killing from the inside: Intracellular role of T3SS in the fate of Pseudomonas aeruginosa within macrophages revealed by mgtC and oprF mutants
Source: PLoS Pathog. 2019 Jun 20;15(6):e1007812. doi: 10.1371/journal.ppat.1007812 (PMC6586356; doi:10.1371/journal.ppat.1007812)
Supplement: S2 Fig — HMDMs were infected with PAO1 wild-type (WT) strain expressing GFP. Time lapse imaging was started at 1.5 hrs post-phagocytosis. Cells were maintained in RPMI supplemented with gentamicin at 37°C and 5% CO2 throughout imaging. White arrows point at the cells that harbor intracellular bacteria and undergo lysis between 1.5 hrs and 3 hrs post-phagocytosis. Black arrow shows an uninfected and unlysed cell. Scale bar is equivalent to 20 μm. (PDF) [file ppat.1007812.s002.pdf]

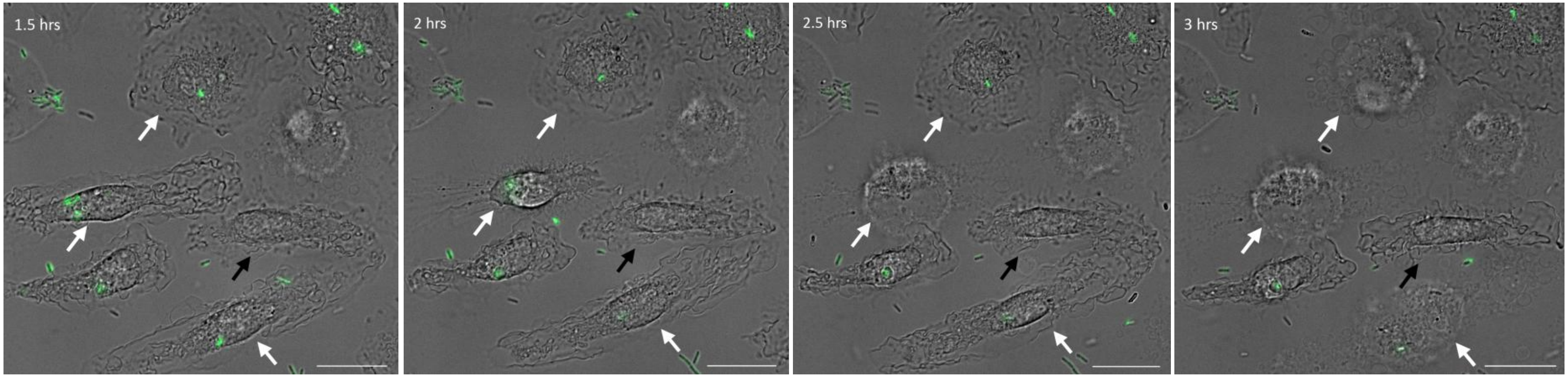

S2 Fig. **Live imaging of primary human macrophages infected with *P. aeruginosa*.** HMDMs were infected with PAO1 wild-type (WT) strain expressing GFP. Time lapse imaging was started at 1.5 hrs post-phagocytosis. Cells were maintained in RPMI supplemented with gentamicin at 37°C and 5% CO_2 throughout imaging. White arrows point at the cells that harbor intracellular bacteria and undergo lysis between 1.5 hrs and 3 hrs post-phagocytosis. Black arrow shows an uninfected and unlysed cell. Scale bar is equivalent to 20 µm.
