# Supplementary material for: Killing from the inside: Intracellular role of T3SS in the fate of Pseudomonas aeruginosa within macrophages revealed by mgtC and oprF mutants
Source: PLoS Pathog. 2019 Jun 20;15(6):e1007812. doi: 10.1371/journal.ppat.1007812 (PMC6586356; doi:10.1371/journal.ppat.1007812)
Supplement: S3 Fig — J774 macrophages were infected with PAO1 expressing GFP. After 2.5 hrs of gentamicin treatment, infected J774 cells were incubated with Lysotracker for 10 min, a red fluorescent weak base that accumulates in acidic compartments. Cells were then fixed and imaged with fluorescence microscope. (A) The image shows individual panels for Differential Interference Contrast (DIC), lysosomal compartment (red), bacteria expressing GFP (green), the nucleus (blue) and merged image of all channels. The solid arrow shows colocalization of bacteria with lysotracker and dashed arrow shows non-colocalization. Scale bar is equivalent to 5 μm. (B) 3D-reconstructed image of the same area of (A). (PDF) [file ppat.1007812.s003.pdf]

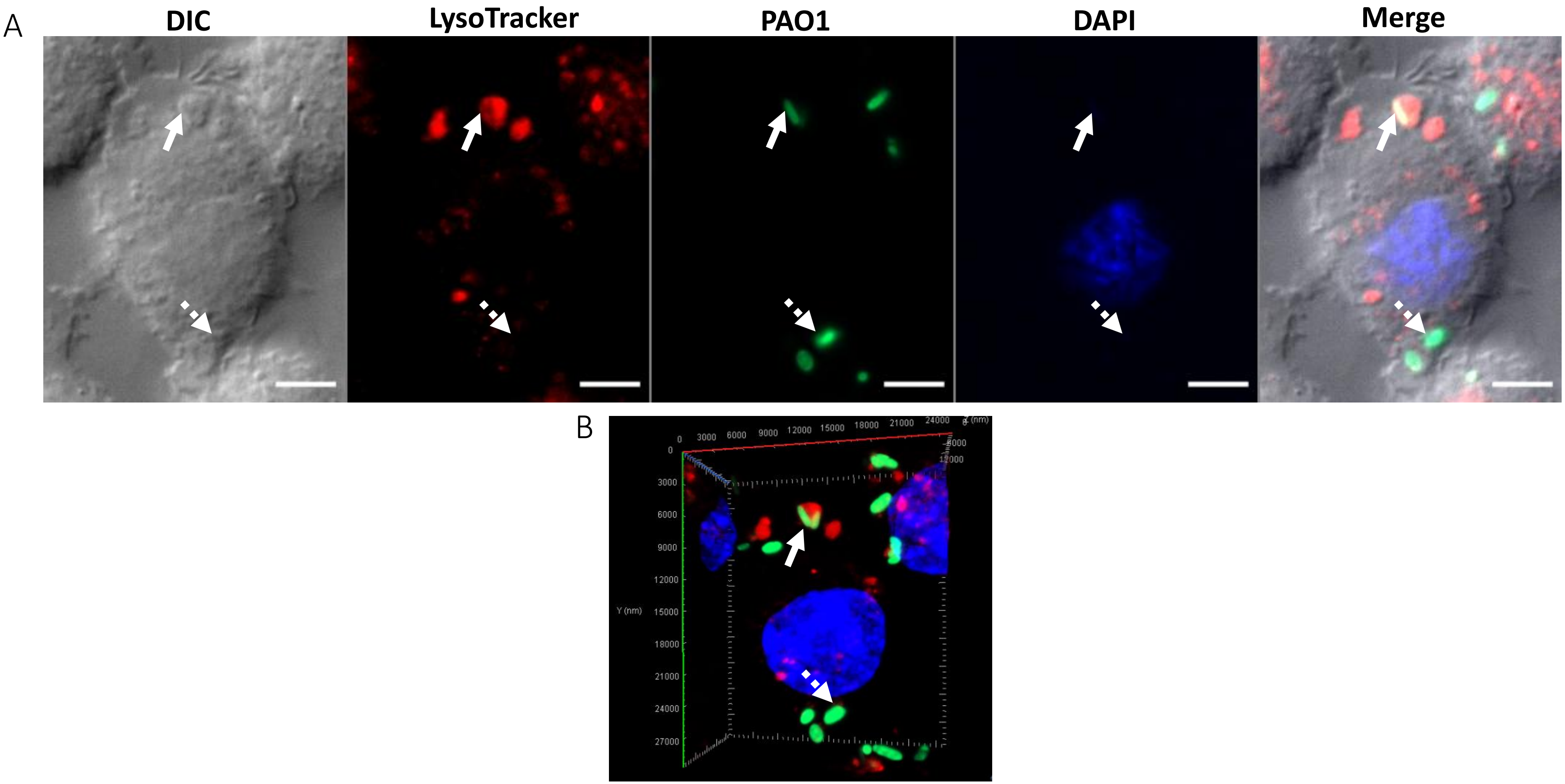

S3 Fig. **Colocalization of *P. aeruginosa* with a probe that labels acidic compartments.** J774 macrophages were infected with PAO1 expressing GFP. After 2.5 hrs of gentamicin treatment, infected J774 cells were incubated with Lysotracker for 10 min, a red fluorescent weak base that accumulates in acidic compartments. Cells were then fixed and imaged with fluorescence microscope. **(A)** The image shows individual panels for Differential Interference Contrast (DIC), lysosomal compartment (red), bacteria expressing GFP (green), the nucleus (blue) and merged image of all channels. The solid arrow shows colocalization of bacteria with lysotracker and dashed arrow shows non-colocalization. Scale bar is equivalent to 5 µm. **(B)** 3D-reconstructed image of the same area of **(A)**.
