# Supplementary material for: Killing from the inside: Intracellular role of T3SS in the fate of Pseudomonas aeruginosa within macrophages revealed by mgtC and oprF mutants
Source: PLoS Pathog. 2019 Jun 20;15(6):e1007812. doi: 10.1371/journal.ppat.1007812 (PMC6586356; doi:10.1371/journal.ppat.1007812)
Supplement: S4 Fig — Visualization (A) and quantification (B) of lysed infected cells upon cytochalasin D treatment. GFP expressing PAO1 was used for infecting J774 macrophages. DMEM containing 2 μM cytochalasin D was added to the macrophages 1 hour before infection and maintained during phagocytosis. After phagocytosis, cells were maintained in 1 μM cytochalasin D in DMEM supplemented with gentamicin till the end of the experiment. 0.2% DMSO in DMEM was added to the cells as solvent control before and during phagocytosis. After phagocytosis, cells were maintained in 0.1% DMSO in DMEM with gentamicin, fixed 2 hrs post-phagocytosis, stained with phalloidin and imaged with fluorescent microscope. DAPI was used to stain the nucleus. Cells that have intracellular bacteria, but lack the phalloidin cortical label, were considered as lysed by intracellular bacteria (shown by arrows). Scale bar is equivalent to 10 μm. Percentage of lysed cells with intracellular bacteria out of total number of cells was plotted. Error bars correspond to standard errors from two independent experiments. At least 200 cells were counted per strain. The asterisks indicate P values (Student’s t-test, **P <0.01), showing statistical significance with respect to DMSO control. (PDF) [file ppat.1007812.s004.pdf]

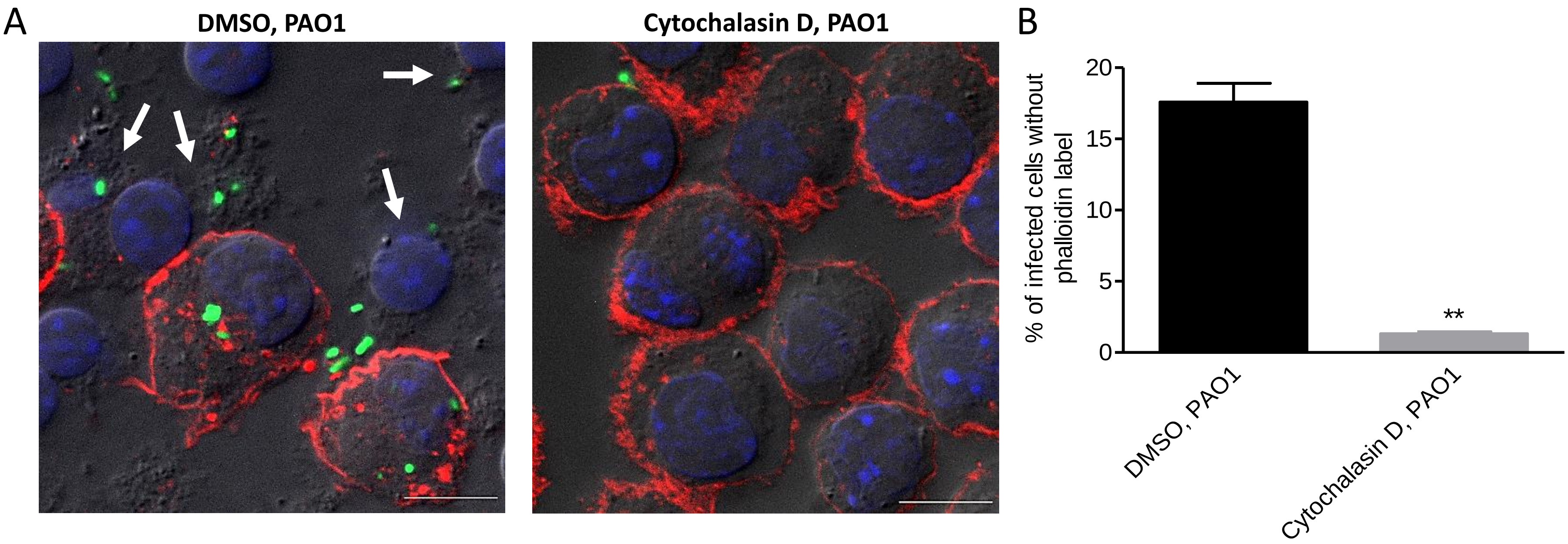

S4 Fig. Visualization (A) and quantification (B) of dying infected cells upon cytochalasin D treatment. GFP expressing PAO1 was used for infecting J774 macrophages. DMEM containing 2 µM cytochalasin D was added to the macrophages 1 hour before infection and maintained during phagocytosis. After phagocytosis, cells were maintained in 1 µM cytochalasin D in DMEM supplemented with gentamicin till the end of the experiment. 0.2 % DMSO in DMEM was added to the cells as solvent control before and during phagocytosis. After phagocytosis, cells were maintained in 0.1 % DMSO in DMEM with gentamicin, fixed 2 hrs post-phagocytosis, stained with phalloidin and imaged with fluorescent microscope. DAPI was used to stain the nucleus. Cells that have intracellular bacteria, but lack the phalloidin cortical label, were considered as lysed by intracellular bacteria (shown by arrows). Scale bar is equivalent to 10 µm. Percentage of lysed cells with intracellular bacteria out of total number of cells was plotted. Error bars correspond to standard errors from two independent experiments. At least 200 cells were counted per strain. The asterisks indicate *P* values (Student's t-test, $^{**}P < 0.01$), showing statistical significance with respect to DMSO control.
