# Supplementary material for: Killing from the inside: Intracellular role of T3SS in the fate of Pseudomonas aeruginosa within macrophages revealed by mgtC and oprF mutants
Source: PLoS Pathog. 2019 Jun 20;15(6):e1007812. doi: 10.1371/journal.ppat.1007812 (PMC6586356; doi:10.1371/journal.ppat.1007812)
Supplement: S5 Fig — PAO1 WT, ΔoprF, PAO1 WT + pexsA and ΔoprF + pexsA strains were used for infecting J774 macrophages. Expression of exsA was induced by adding 0.01 mM IPTG for the strains PAO1 WT + pexsA and ΔoprF + pexsA. Gentamicin was added after phagocytosis and cells were fixed at 2 hrs post-phagocytosis, stained with phalloidin and imaged with fluorescent microscope. DAPI was used to stain the nucleus. Cells that lacked the phalloidin cortical label were considered as lysed by intracellular bacteria. After imaging, percentage of lysed cells out of total number of cells was plotted for each strain. Error bars correspond to standard errors from at least three independent experiments. At least 200 cells were counted per strain. The asterisks indicate P values (One way ANOVA, where all strains were compared to each other using Bonferroni’s multiple comparison post-test, **P <0.01 and ns = P >0.05), showing statistical significance with respect to WT. (PDF) [file ppat.1007812.s005.pdf]

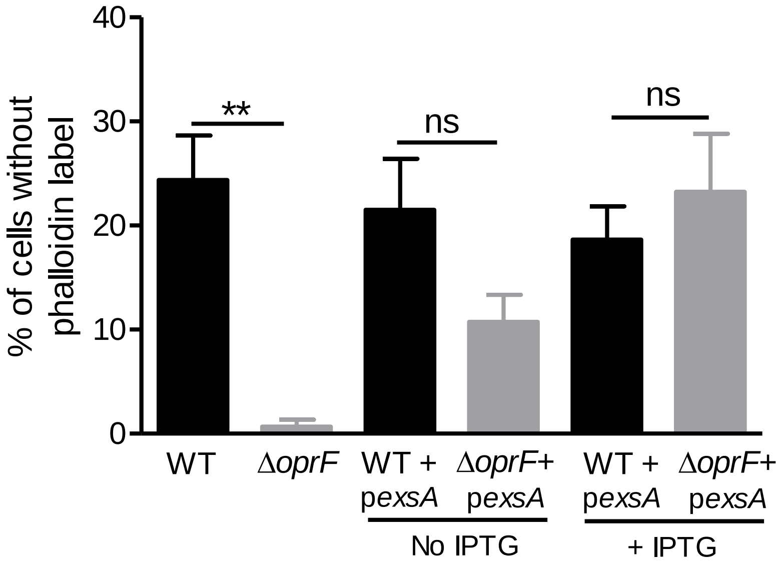

S5 Fig. Q**uantification of dying cells infected with *P. aeruginosa* strains overexpressing *exsA*.** PAO1 WT, Δ*oprF*, PAO1 WT + p*exsA* and Δ*oprF* + p*exsA* strains were used for infecting J774 macrophages. Expression of *exsA* was induced by adding 0.01 mM IPTG for the strains PAO1 WT + p*exsA* and Δ*oprF* + p*exsA*. Gentamicin was added after phagocytosis and cells were fixed at 2 hrs post-phagocytosis, stained with phalloidin and imaged with fluorescent microscope. DAPI was used to stain the nucleus. Cells that lacked the phalloidin cortical label were considered as lysed by intracellular bacteria. After imaging, percentage of lysed cells out of total number of cells was plotted for each strain. Error bars correspond to standard errors from at least three independent experiments. At least 200 cells were counted per strain. The asterisks indicate *P* values (One way ANOVA, where all strains were compared to each other using Bonferroni's multiple comparison post-test, $^{**}P < 0.01$ and ns = $P > 0.05$), showing statistical significance with respect to WT.
