# Supplementary material for: Killing from the inside: Intracellular role of T3SS in the fate of Pseudomonas aeruginosa within macrophages revealed by mgtC and oprF mutants
Source: PLoS Pathog. 2019 Jun 20;15(6):e1007812. doi: 10.1371/journal.ppat.1007812 (PMC6586356; doi:10.1371/journal.ppat.1007812)
Supplement: S6 Fig — Release of LDH was measured from J774 macrophages infected for 2 hrs with PAO1 WT, ΔpscN, ΔexoS and ΔexoSTY strains to quantify the cytotoxicity. The percentage of LDH release was calculated relatively to that of total uninfected cells lysed with Triton X-100, which was set at 100% LDH release. Error bars correspond to standard errors (SE) from at least four independent experiments. The asterisks indicate P values (One way ANOVA, where all strains were compared to WT using Dunnett’s multiple comparison post-test, **P <0.01), showing statistical significance with respect to WT. (PDF) [file ppat.1007812.s006.pdf]

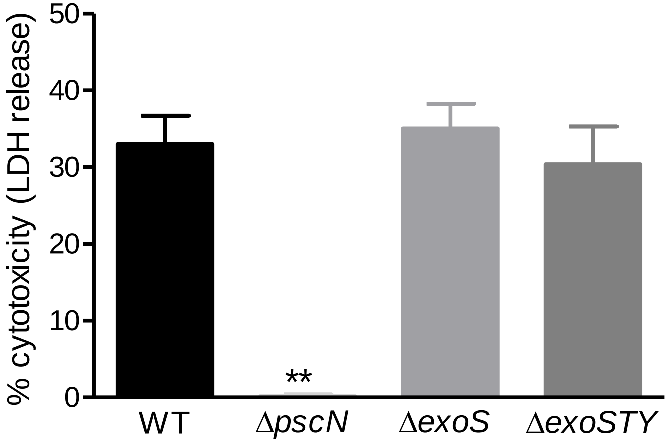

S6 Fig. Quantification of cell lysis driven by extracellular bacteria. Release of LDH was measured from J774 macrophages infected for 2 hrs with PAO1 WT, Δ*pscN*, Δ*exoS* and Δ*exoSTY* strains to quantify the cytotoxicity. The percentage of LDH release was calculated relatively to that of total uninfected cells lysed with Triton X-100, which was set at 100% LDH release. Error bars correspond to standard errors (SE) from at least four independent experiments. The asterisks indicate *P* values (One way ANOVA, where all strains were compared to WT using Dunnet's multiple comparison post-test, ***P* <0.01), showing statistical significance with respect to WT.
