# Supplementary material for: Killing from the inside: Intracellular role of T3SS in the fate of Pseudomonas aeruginosa within macrophages revealed by mgtC and oprF mutants
Source: PLoS Pathog. 2019 Jun 20;15(6):e1007812. doi: 10.1371/journal.ppat.1007812 (PMC6586356; doi:10.1371/journal.ppat.1007812)
Supplement: S7 Fig — J774 macrophages were infected with the strains as indicated. After phagocytosis, cells were maintained in DMEM supplemented with gentamicin. Cells were stained with trypan blue at (A) 30 min or (B) 2 hrs post-phagocytosis and imaged. Lysed cells were quantified by counting cells stained with trypan blue and the percentage of lysed cells out of total number of cells was plotted. Error bars correspond to standard errors from at least three independent experiments. At least 400 cells were counted per strain. The asterisks indicate P values (One way ANOVA, where all strains were compared to WT using Dunnett’s multiple comparison test, *P <0.05, **P <0.01 and ***P <0.001), showing statistical significance with respect to WT. (PDF) [file ppat.1007812.s007.pdf]

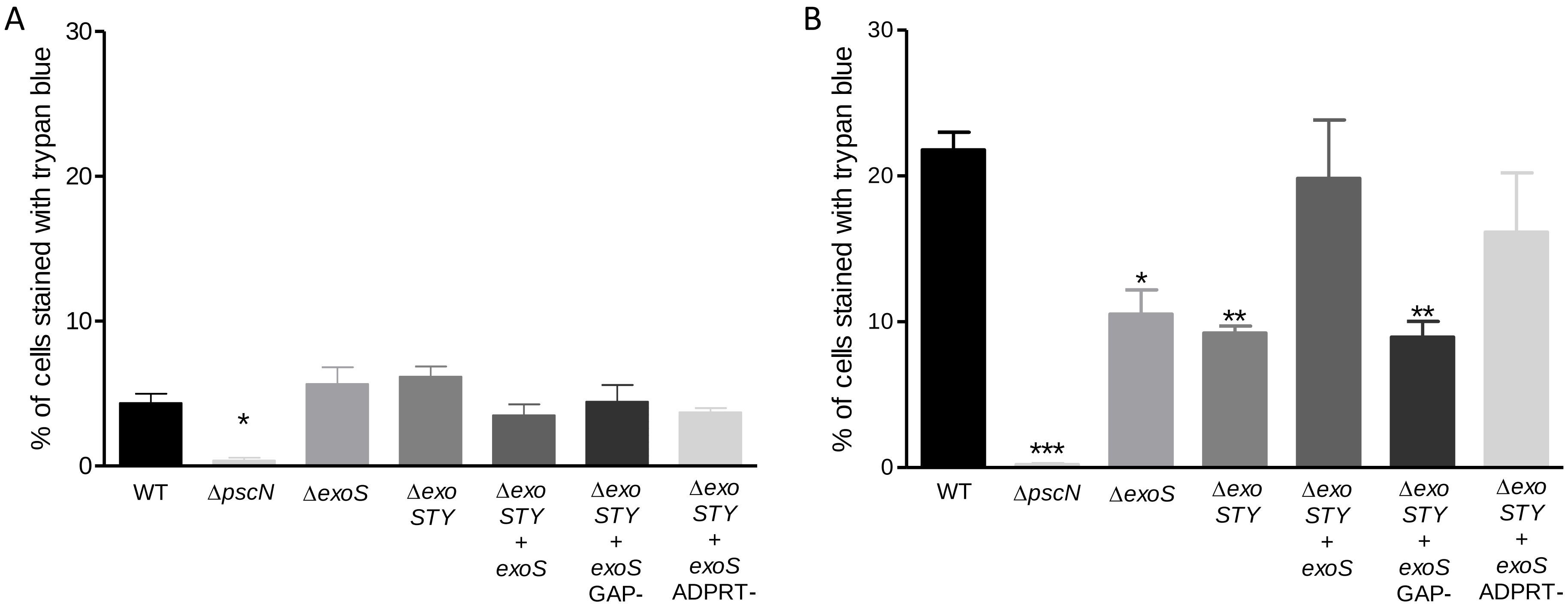

S7 Fig. Quantification of lysed cells by staining with Trypan Blue. J774 macrophages were infected with the strains as indicated. After phagocytosis, cells were maintained in DMEM supplemented with gentamicin. Cells were stained with trypan blue at (A) 30 min or (B) 2 hrs post-phagocytosis and imaged. Lysed cells were quantified by counting cells stained with trypan blue and the percentage of lysed cells out of total number of cells was plotted. Error bars correspond to standard errors from at least three independent experiments. At least 400 cells were counted per strain. The asterisks indicate *P* values (One way ANOVA, where all strains were compared to WT using Dunnet's multiple comparison test, **P* <0.05, ***P* <0.01 and ****P* <0.001), showing statistical significance with respect to WT.
