# Supplementary material for: Killing from the inside: Intracellular role of T3SS in the fate of Pseudomonas aeruginosa within macrophages revealed by mgtC and oprF mutants
Source: PLoS Pathog. 2019 Jun 20;15(6):e1007812. doi: 10.1371/journal.ppat.1007812 (PMC6586356; doi:10.1371/journal.ppat.1007812)
Supplement: S8 Fig — PAO1 WT, ΔmgtC, ΔoprF, ΔpscN, ΔexoS and ΔexoSTY strains grown in presence of ampicillin, were incubated with CCF4-AM for 1 hour. The blue fluorescence generated as a result of loss of FRET of CCF4 was measured (excitation, 420 nm and emission, 450 nm) and plotted as arbitrary units (AU). Error bars correspond to standard errors from four independent experiments. All strains were compared to WT using One way ANOVA, Dunnett’s multiple comparison post-test. No significant difference was found between the mutant strains and WT. (PDF) [file ppat.1007812.s008.pdf]

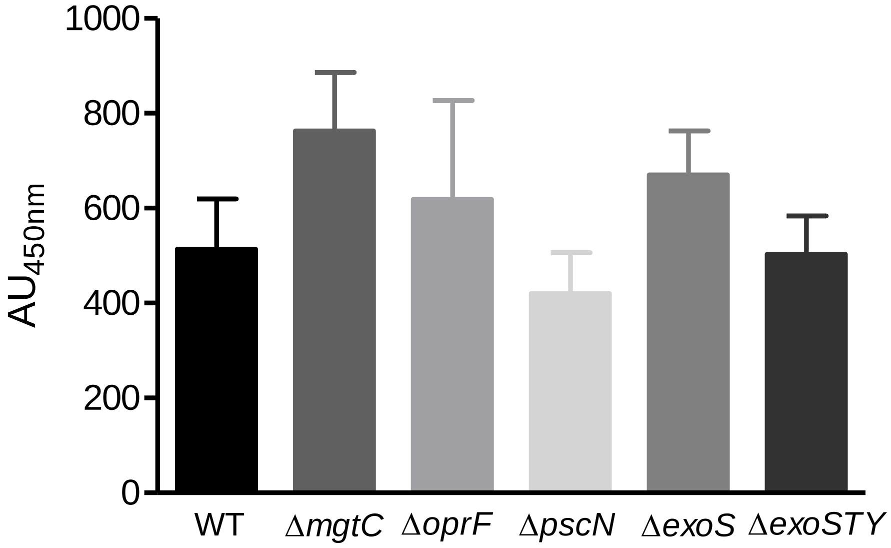

S8 Fig. Assessment of β-lactamase activity of *P. aeruginosa* strains in liquid culture. PAO1 WT, Δ*mgtC*, Δ*oprF*, Δ*pscN*, Δ*exoS* and Δ*exoSTY* strains, grown in presence of ampicillin, were incubated with CCF4-AM for 1 hour. The blue fluorescence generated as a result of loss of FRET of CCF4 was measured (excitation, 420 nm and emission, 450 nm) and plotted as arbitrary units (AU). Error bars correspond to standard errors from four independent experiments. All strains were compared to WT using One way ANOVA, Dunnet's multiple comparison post-test. No significant difference was found between the mutant strains and WT.
