# Supplementary material for: Killing from the inside: Intracellular role of T3SS in the fate of Pseudomonas aeruginosa within macrophages revealed by mgtC and oprF mutants
Source: PLoS Pathog. 2019 Jun 20;15(6):e1007812. doi: 10.1371/journal.ppat.1007812 (PMC6586356; doi:10.1371/journal.ppat.1007812)
Supplement: S9 Fig — J774 macrophages were infected with PAO1 WT, ΔpscN, ΔexoS and ΔexoSTY strains. After phagocytosis, cells were stained with CCF4-AM in presence of gentamicin. 2 hrs post-phagocytosis, the cells were imaged with 10X objective using FITC and DAPI channels. Upon escape of bacteria from phagosome to the cytosol, the CCF4-AM FRET is lost, producing blue color. Images were analyzed and quantified by Cell Profiler software to calculate the percentage of blue cells out of total green cells. At least 200 cells were counted per strain. Error bars correspond to standard errors from four independent experiments. The asterisks indicate P values (One way ANOVA, where all strains were compared to WT using Dunnett’s multiple comparison post-test, *P <0.05), showing statistical significance with respect to WT. (PDF) [file ppat.1007812.s009.pdf]

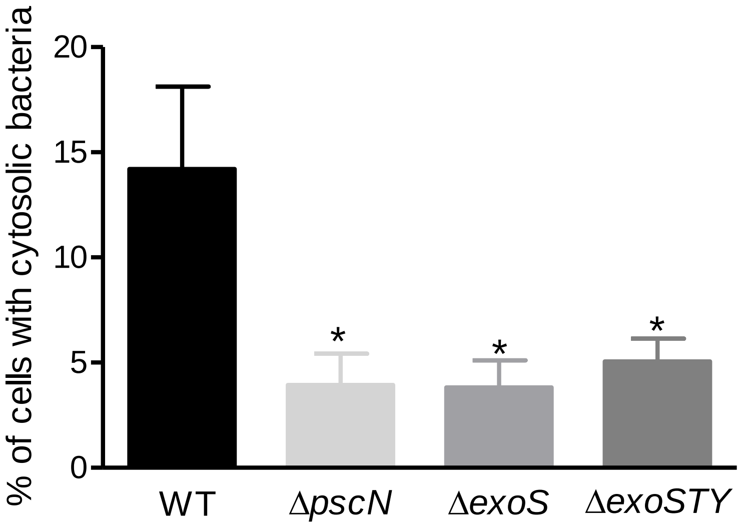

S9 Fig. Phagosome escape assay of T3SS mutants. J774 macrophages were infected with PAO1 WT, Δ*pscN*, Δ*exoS* and Δ*exoSTY* strains. After phagocytosis, cells were stained with CCF4-AM in presence of gentamicin. 2 hours post-phagocytosis, the cells were imaged with 10X objective using FITC and DAPI channels. Upon escape of bacteria from phagosome to the cytosol, the CCF4-AM FRET is lost, producing blue color. Images were analyzed and quantified by Cell Profiler software to calculate the percentage of blue cells out of total number of green cells. At least 200 cells were counted per strain. Error bars correspond to standard errors from four independent experiments. The asterisks indicate *P* values (One way ANOVA, where all strains were compared to WT using Dunnet's multiple comparison post-test , *P <0.05), showing statistical significance with respect to WT.
