# Supplementary material for: Killing from the inside: Intracellular role of T3SS in the fate of Pseudomonas aeruginosa within macrophages revealed by mgtC and oprF mutants
Source: PLoS Pathog. 2019 Jun 20;15(6):e1007812. doi: 10.1371/journal.ppat.1007812 (PMC6586356; doi:10.1371/journal.ppat.1007812)
Supplement: S10 Fig — Immunodetection of the ExoS effector in culture supernatant of PAO1 and PAO1 H103 (isogenic WT strain for oprF mutant) grown in exponential phase at 37°C under T3SS-inducing (− Ca2+) or non-inducing (+ Ca2+) conditions. The bacterial culture supernatant (equivalent of 1 OD600 unit) was loaded on an SDS gel containing 10% polyacrylamide. As control, cellular fraction (equivalent of 0.1 OD600 unit) was loaded. Upper panels show Western blot using anti-ExoS antibodies (Soscia et al., 2007; doi: 10.1128/JB.01677-06) and lower panels show Coomassie stained gels. (PDF) [file ppat.1007812.s010.pdf]

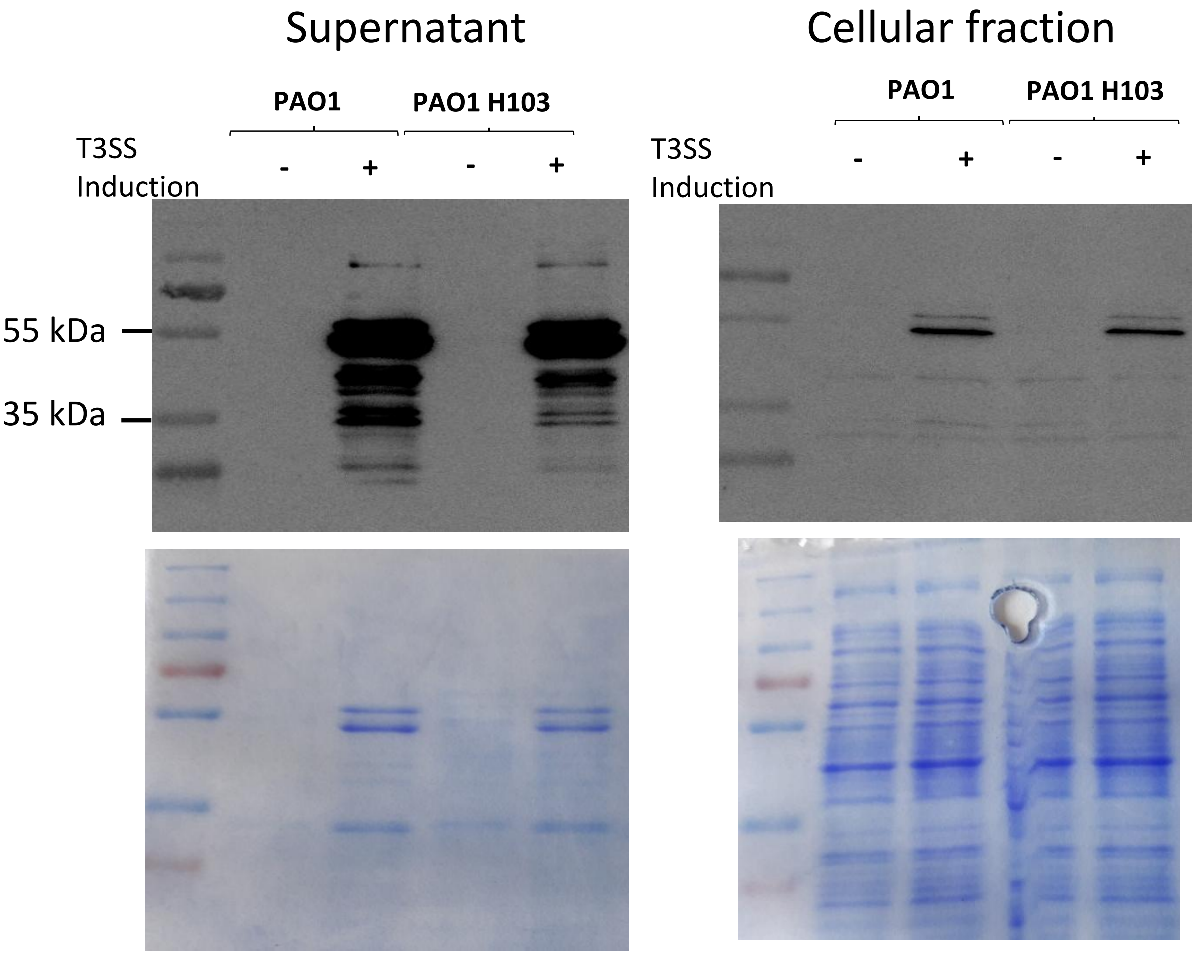

S10 Fig. Comparison of secreted protein profiles and ExoS production under T3SS-inducing conditions in two PAO1 strains. Immunodetection of the ExoS effector in culture supernatant of PAO1 and PAO1 H103 (isogenic WT strain for *oprF* mutant) grown in exponential phase at 37°C under T3SS-inducing (− Ca^{2+}) or non-inducing (+ Ca^{2+}) conditions. The bacterial culture supernatant (equivalent of 1 OD_{600} unit) was loaded on an SDS gel containing 10% polyacrylamide. As control, cellular fraction (equivalent of 0.1 OD_{600} unit) was loaded. Upper panels show Western blot using anti-ExoS antibodies (Soscia et al., 2007; doi: 10.1128/JB.01677-06) and lower panels show Coomassie stained gels.
