# Supplementary material for: Killing from the inside: Intracellular role of T3SS in the fate of Pseudomonas aeruginosa within macrophages revealed by mgtC and oprF mutants
Source: PLoS Pathog. 2019 Jun 20;15(6):e1007812. doi: 10.1371/journal.ppat.1007812 (PMC6586356; doi:10.1371/journal.ppat.1007812)
Supplement: S1 Table — (PDF) [file ppat.1007812.s012.pdf]

S1 Table. List of primers used for RT-PCR.

RT primers	Sequence
***rpoD*-RT-fwd**	5’-GGGCGAAGAAGGAAATGGTC-3’
***rpoD*-RT-rev**	5’-CAGGTGGCGTAGGTGGAGAA-3’
***exoS*-RT-fwd**	5’-GCCTTGTCGAGTCCCTTCAA-3’
***exoS*-RT-rev**	5’-CTTCGTGGCGATCATGGACT-3’
***pcrV*-RT-fwd**	5’-TGGGTCTGCAGGACATCCTT-3’
***pcrV*-RT-rev**	5’-AACGACTGGAACAAGCCCTAC-3’
***fliC*-RT-fwd**	5’-CCTGCAGAAAGAAGTCGCTG-3’
***fliC*-RT-rev**	5’-GTTGCTGCCGACCTGGTAA-3’
